# Open femoral hernia repair: one skin incision for all

**DOI:** 10.1186/1749-7922-4-44

**Published:** 2009-11-30

**Authors:** Paolo G Sorelli, Nabil S El-Masry, William V Garrett

**Affiliations:** 1 Department of GI Surgery, Medway Maritime Hospital, King's College, UK; 2 Department of GI Surgery, Charing Cross Hospital, UK

## Abstract

**Background:**

Femoral hernias are relatively uncommon, however they are the most common incarcerated abdominal hernia, with strangulation of a viscus carrying significant mortality. Classically three approaches are described to open femoral hernia repair: Lockwood's infra-inguinal, Lotheissen's trans-inguinal and McEvedy's high approach. Each approach describes a separate skin incision and dissection to access the femoral sac. The decision as to which approach to adopt, predominantly dependent on the suspicion of finding strangulated bowel, is often a difficult one and in our opinion an unnecessary one.

**Methods:**

We propose a technique for open femoral hernia repair that involves a single skin incision 1 cm above the medial half of the inguinal ligament that allows all of the above approaches to the hernia sac depending on the operative findings. Thus the repair of simple femoral hernias can be performed from below the inguinal ligament. If found, inguinal hernias can be repaired. More importantly, resection of compromised bowel can be achieved by accessing the peritoneal cavity with division of the linea semilunaris 4 cm above the inguinal ligament. This avoids compromise of the inguinal canal, and with medial retraction of the rectus abdominis muscle enables access to the peritoneal cavity and compromised bowel.

**Discussion:**

This simple technique minimises the preoperative debate as to which incision will allow the best approach to the femoral hernia sac, allow for alteration to a simple inguinal hernia repair if necessary, and more importantly obviate the need for further skin incisions if compromised bowel is encountered that requires resection.

## Background

Femoral hernias are relatively uncommon, making up 2-8% of all adult groin hernias[1,2]. Incarcerated femoral hernias, however, are the most common incarcerated abdominal hernia[3], with strangulation of a viscus carrying up to 14% mortality[4]. Femoral hernias are a common cause of small bowel obstruction and remain the most frequent cause of strangulation in this setting, necessitating immediate operative intervention[5].

Classically three approaches are described to open femoral hernia repair: Lockwood's infra-inguinal approach, Lotheissen's trans-inguinal approach and McEvedy's high approach.

The infra-inguinal approach is the preferred method for elective repair, approaching the femoral canal from below through an oblique incision 1 cm below and parallel to the inguinal ligament. This approach however offers little scope for resecting any compromised bowel.

The trans-inguinal approach involves a skin incision 2 cm above the inguinal ligament, dissecting through the inguinal canal and thus weakening this important structure. The danger with this, particularly in the presence of wound infection, is that a hernia may form later which would be difficult to repair. In addition, if necrotic bowel is encountered the risk of infection may preclude the use of synthetic mesh to repair the inguinal canal and predispose to inguinal hernia occurrence.

The high approach involves an oblique skin incision 3 cm above the pubic tubercle running laterally to cross the lateral border of the rectus muscle, that is divided allowing preperitoneal dissection of the sac. This approach is preferred in the emergency setting when strangulation is suspected allowing better access to and visualisation of bowel for possible resection.

Because of the tendency of femoral hernias to move upward to a position above the inguinal ligament, it may sometimes be mistaken for an inguinal hernia and the correct diagnosis often made only at operation. Frequently the origin of an incarcerated mass may be indistinguishable on physical examination. The presence or absence of compromised sac content is another clinical feature that is often very difficult to predict. In practice therefore these uncertainties make the decision as to which approach to adopt a very difficult one, and in our opinion an unnecessary one.

## Method

We propose a technique for open femoral hernia repair that involves a single skin incision through which all of the above approaches to the sac can be undertaken depending on the operative findings.

**Figure 1:**
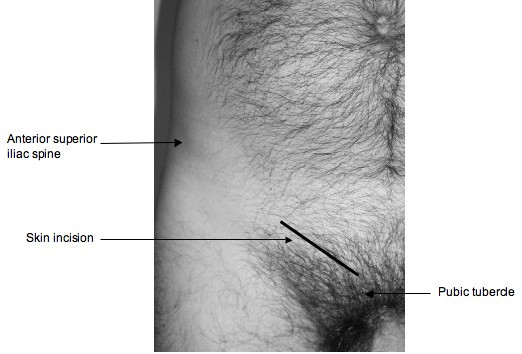
**Surface anatomy and skin incision**.

**Figure 2:**
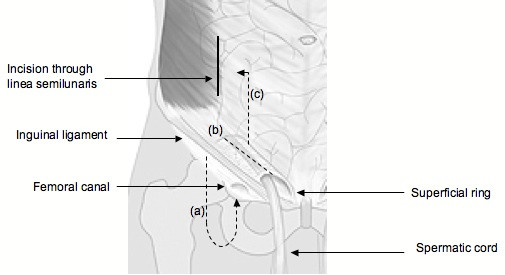
**Approaches to the hernial sac**: (a) Infrainguinal approach. (b) Transinguinal approach. (c) High approach.

Through an inguinal incision, 1 cm above the medial half of the inguinal ligament (Figure 1) the femoral hernia sac can be explored from below (Lockwood approach) (Figure 2 - (a)). A simple femoral hernia repair can then be performed if this is found without compromised sac content.

If no femoral hernia is discovered but an inguinal hernia is present, then the inguinal canal can be explored by dividing the external oblique aponeurosis (Figure 2 - (b)) and completing a classical open inguinal hernia repair of the surgeon's choice.

More importantly however, with this technique, if the femoral hernia contains compromised bowel requiring resection, this can be achieved by creating a plane superficial to the external oblique aponeurosis (Figure 2 - (c)). The rectus sheath is then divided along the linea semilunaris 4 cm above the inguinal ligament (Figure 2), thus preserving the inguinal canal, but exposing the lateral border of the rectus abdominis muscle which is retracted medially. Then the fascia transversalis and peritoneum are divided giving access to the peritoneal cavity and compromised bowel.

## Discussion

We do not presume to be the first to have performed this technique, but we are not aware that it has been formally reported in the literature. More importantly surgical teaching is still centred around the three classical approaches to femoral hernia repair, and, although we do not deny the historical value of these, we feel that awareness of this approach is of value for the surgical trainee.

Although rare, we estimate that we perform approximately 3-4 emergency femoral hernia repairs per year using this technique, and to date collaboratively have performed 78 cases. There have been no complications associated with this technique although we do not suggest that complications associated with any groin hernia operation such as seroma formation and wound infection are significantly decreased with this approach. We are not aware of any hernia recurrences using this technique although the age group and co-morbidities of the patients involved often preclude long term follow up, as do restrictions on clinic space in the current NHS. In terms of post-operative recovery, particularly where strangulated bowel is encountered, the lack of conversion to laparotomy or further skin incisions can only, we believe, contribute to quicker recovery times.

Most importantly however, this simple technique minimises the preoperative debate as to which incision will allow the best approach to the femoral sac, allow for alteration to a simple inguinal hernia repair if necessary, and more importantly obviate the need for further skin incisions if compromised bowel is encountered that requires resection.

## Competing interests

The authors declare that they have no competing interests.

## Authors' contributions

All authors have contributed fully to 1) conception and design of the manuscript 2) drafting the manuscript and 3) final approval of the version to be published.
